# Methyl Salicylate Glycosides in Some Italian Varietal Wines

**DOI:** 10.3390/molecules24183260

**Published:** 2019-09-06

**Authors:** Silvia Carlin, Domenico Masuero, Graziano Guella, Urska Vrhovsek, Fulvio Mattivi

**Affiliations:** 1 Department of Food Quality and Nutrition, Research and Innovation Centre, Fondazione Edmund Mach (FEM), Via E. Mach, 1 38010 S. Michele all’Adige, TN, Italy; 2 Department of Agricultural, Food, Environmental and Animal Sciences, University of Udine, Via delle Scienze 208, 33100 Udine, Italy; 3 Bioorganic Chemistry Laboratory, Department of Physics, University of Trento, 38123 Trento, Italy

**Keywords:** methyl salicylate, glycosides, Verdicchio wine, gaultherin, violutoside, methyl salicylate glucoside, methyl salicylate canthoside A, methyl salicylate gentiobioside, methyl salicylate rutinoside, methyl salicylate sambubioside

## Abstract

Glycosides are ubiquitous plant secondary metabolites consisting of a non-sugar component called an aglycone, attached to one or more sugars. One of the most interesting aglycones in grapes and wine is methyl salicylate (MeSA), an organic ester naturally produced by many plants, particularly wintergreens. To date, nine different MeSA glycosides from plants have been reported, mainly spread over the genera *Gaultheria*, *Camellia*, *Polygala*, *Filipendula*, and *Passiflora*. From a sensorial point of view, MeSA has a balsamic-sweet odor, known as Wintergreen. MeSA was found in *Vitis riparia* grapes, in *Vitis vinifera* sp. and in the Frontenac interspecific hybrid. We found that the MeSA glycosides content in Verdicchio wines and in some genetically related varieties (Trebbiano di Soave and Trebbiano di Lugana) was very high. In order to understand which glycosides were present in wine, the methanolic extract of Verdicchio wine was injected into a UPLC-Q-TOF-HDMS and compared to the extracts of different plants rich in such glycosides. Using pure standards, we confirmed the existence of two glycosides in wine: MeSA 2-*O*-β-d-glucoside and MeSA 2-*O*-β-d-xylopyranosyl (1-6) β-d-glucopyranoside (gaultherin). For the first time, we also tentatively identified other diglycosides in wine: MeSA 2-*O*-α-l-arabinopyranosyl (1-6)-β-d-glucopyranoside (violutoside) and MeSA 2-*O*-β-d-apiofuranosyl (1-6)-β-d-glucopyranoside (canthoside A), MeSA 2-*O*-β-d-glucopyranosyl (1-6)-*O*-β-d-glucopyranoside (gentiobioside) and MeSA 2-*O*-α-l-rhamnopyranosyl (1-6)-β-d-glucopyranoside (rutinoside). Some of these glycosides have been isolated from *Gaultheria procumbens* leaves by preparative liquid chromatography and structurally annotated by ^1^H- and ^13^C-NMR analysis. Two of the peaks isolated from *Gaultheria procumbens* leaves, namely MeSA sambubioside and MeSA sophoroside, were herein observed for the first time. Six MeSA glycosides were quantified in 64 Italian white wines, highlighting the peculiar content and pattern in Verdicchio wines and related cultivars. The total concentration in bound and free MeSA in Verdicchio wines varied in the range of 456–9796 μg/L and 5.5–143 μg/L, respectively, while in the other wines the bound and free MeSA was below 363 μg/L and 12 μg/L, respectively. As this compound’s olfactory threshold is between 50 and 100 μg/L, our data support the hypothesis that methyl salicylate can contribute to the balsamic scent, especially in old Verdicchio wines.

## 1. Introduction

Glycosides are plant secondary metabolites consisting of a non-sugar component called an aglycone, attached to one or more sugars. Glycosides are ubiquitous in the plant kingdom and are present in all plant organs such as fruit, flowers, roots, seed, and bark [1]. Most aglycones are non-polar and glycosylation increases their water solubility and facilitates their transport, accumulation, and storage, together with the detoxification of some of these compounds [2,3]. The aglycone can be an aliphatic alcohol (C6 compounds), a shikimate derivate (benzyl alcohol, phenols, or methyl salicylate) or terpenoid (monoterpenoid or norisoprenoid). All of these compounds can be a reserve of odor compounds after hydrolysis [4]. While the glycosides of terpenes and norisoprenoids present in wine have been the subject of many studies [5,6], those of shikimate derivate are less considered. These are particularly important especially for neutral varieties, since they have been shown to significantly contribute to the dry aromas of figs, tobacco, and chocolate in some wines [7].

Hydrolysis of these “bound” compounds occurs during fermentation or storage. The most important factors that increase this reaction are enzymatic activity during fermentation, pH/temperature during storage, and, of course, the amount of precursors in the grapes. It is well-known that the monoterpenes released from glycosides during winemaking and aging are important for the fruity and floral flavor of wines such as Muscat and Gewürztraminer [8]. The glycosides of C13-norisoprenoids are more important in other wines, with tea and grassy notes in Chardonnay and Semillon wines [9], but also kerosene-like characteristics in aged Riesling wine.

Monoterpene glycosides release volatile aroma compounds directly via hydrolysis, while norisoprenoid glycosides may release odorless products after hydrolysis which require other chemical reactions to produce volatile aroma compounds [1,10]. Glycosides in wine originate from the grape berry during ripening and appear to be correlated with the concentration of their corresponding aglycones. A recent study demonstrated that grapevine exposure to bushfire smoke can lead to an accumulation in the berries of exogenous volatile phenols in glycoconjugate forms which can also be released in their free form during winemaking [11]. The aglycone is always attached directly to β-d-glucose, and the glucose can be further substituted by other sugars such as α-l-arabinofuranose, α-l-rhamnopyranose, β-d-xylopyranose, β-d-apiofuranose, and β-d-glucose to give the corresponding disaccharides. Rhamnosyl-glucoside is commonly called rutinoside, glucosyl-glucoside, gentiobioside or sophoroside, depending on a 2-*O* rather than 6-*O* linkage [12]. Due to their potential role in the aroma characteristics of wine, the quantification of these precursors could be useful for winemakers to determine, for instance, the optimal maturity of grapes and the most suitable winemaking processes to best enhance them. One of the aglycones found in grapes and wine is methyl salicylate. The methyl salicylate group could be linked with the –OH of a glucopyranosyl unit, and glucose could be further substituted by other sugars such as α-l-arabinofuranose, α-l-rhamnopyranose, β-d-xylopyranose, β-d-apiofuranose, and β-d-glucose to give the corresponding disaccharides. Some of these glycosides are expected to substitute for aspirin due to their long-term effects and fewer side effects. To date, nine different methyl salicylate glycosides from plants have been reported [13]. These methyl salicylate glycosides are mainly spread over the genera *Gaultheria, Camellia, Polygala, Filipendula,* and *Passiflora*. Some of these plants have been used in traditional medicine for centuries. Methyl salicylate was found in *V. riparia* grapes by [14], in *V. vinifera* sp. [15,16], and in the Frontenac interspecific hybrid [17]. Recently, the importance of methyl salicylate as a key aroma released by precursors has been observed in Verdicchio wines [18]. The existence of two glycosides was hypothesized in a previous study carried out in our laboratory: methyl salicylate 2-*O*-β-d-glucoside and methyl salicylate 2-*O*-β-d-xylopyranosyl (1-6) β-d-glucopyranoside (MeSA-primeveroside or gaultherin). This study demonstrated that methyl salicylate glycoside content in Verdicchio, Trebbiano di Soave, and Trebbiano di Lugana wines was very high in comparison to other varieties (up to 10 mg/L). All of these varieties are genetically similar [19]. Since this compound’s olfactory threshold is between 50 and 100 μg/L, methyl salicylate could potentially contribute to the balsamic scent in Verdicchio aged wines [18]. Glycosidic fraction analysis is usually performed after isolation using solid phase extraction (SPE) and then after acid or enzymatic hydrolysis analysis of the aglycones using GC-MS [20]. To study the precursors in their natural form, some studies were carried out using GC-MS analysis of the trimethylsilyl (TMS) and trifluoroacetyl (TFA) derivatives of terpene glycosides [16,21]) Glycosides can also be analyzed using LC-MS, NMR, and IR [22,23]. In the present study, we decided to identify and quantify the precursors of methyl salicylate in wines in their native glycosidic forms (Table 1) using a liquid chromatography coupled with mass spectrometry.

## 2. Results and Discussion

**Figure 1:**
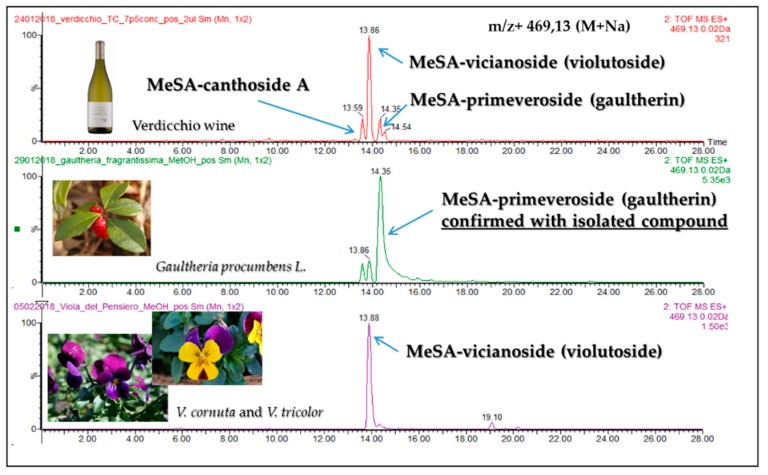
Extracted chromatogram of *m*/*z* 469.13 with the 3 diglycoside isomers found in Verdicchio wine.

The methanolic extract of Verdicchio wine after SPE was injected into a UHPLC coupled to a high resolution mass spectrometer. Six possible MeSA glycosides were found based on the compounds reported in the literature. First, the presence of methyl salicylate monoglycoside (MeSAG) ([M + Na]^+^ peak at *m*/*z* 337.0900 Da) could be observed, confirmed by both the chromatographic and MS data of the commercially available standard. This compound, a methyl salicylate group linked with the –OH of a glucopyranosyl unit, is the structural foundation of all glycosides. We also observed 3 diglycoside isomers ([M + Na]^+^ peak at *m*/*z* 469.1322 Da), (Figure 1), which have the structural unit of MeSAG connected to another monosaccharide that could be apiose/xylose/arabinose. One of these corresponded to gaultherin (glucose + xylose), confirmed by both the chromatographic and MS data of the commercially available standard. In order to identify the other two compounds (glucose + apiose, glucose + arabinose), we tried to extract some glycosides from different plants particularly, in accordance with the literature (Table 1) [13]. The methanolic extracts of two Viola species (*V. cornuta* and *V. tricolor*) which are the richest in MeSA-vicianoside (violutoside glucose + arabinose) were analyzed, and a major peak with mass *m*/*z* 469.1322 (M + Na)^+^, corresponding to MeSA-vicianoside (violutoside), was found. The wine chromatogram (Figure 1) makes it possible to deduce that the second isomer with RT 13.86 should be MeSA-vicianoside (violutoside) with mass *m*/*z* 469.13 (M + Na)^+^ and the first isomer with a retention time of 13.59 could be Canthoside A (glucose + apiose). It is also evident that the most abundant peak in wine is MeSA-vicianoside (violutoside).

**Figure 2:**
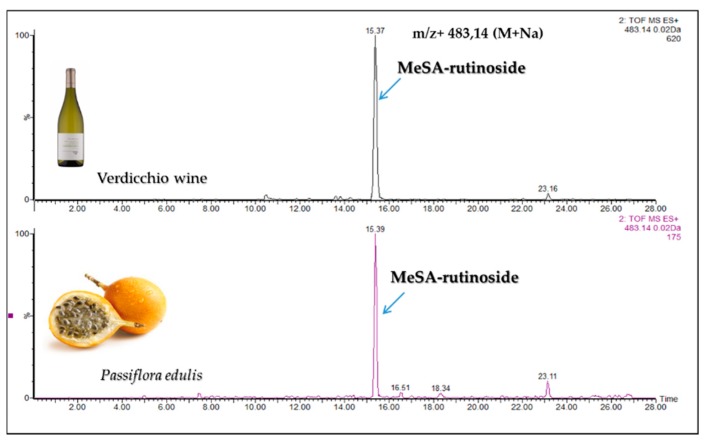
Extracted chromatogram of *m*/*z* 483.14 with rutinoside diglycoside found in Verdicchio wine.

**Figure 3:**
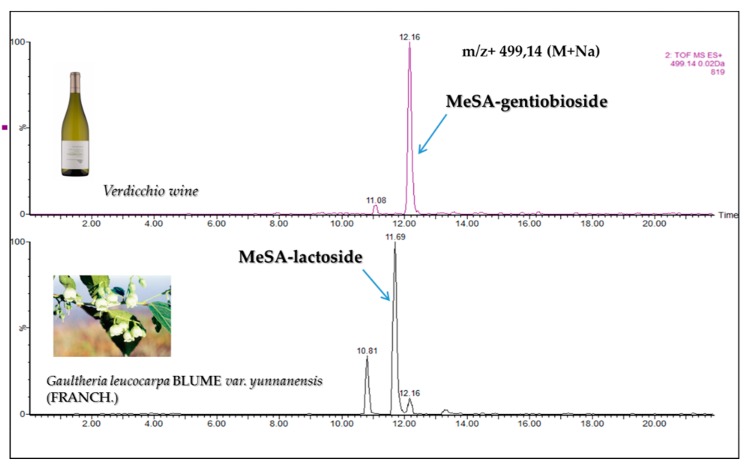
Extracted chromatogram of *m*/*z* 499.14 with the 2 diglycoside isomers. MeSA-gentiobioside is the only one present in Verdicchio wine.

**Figure 4:**
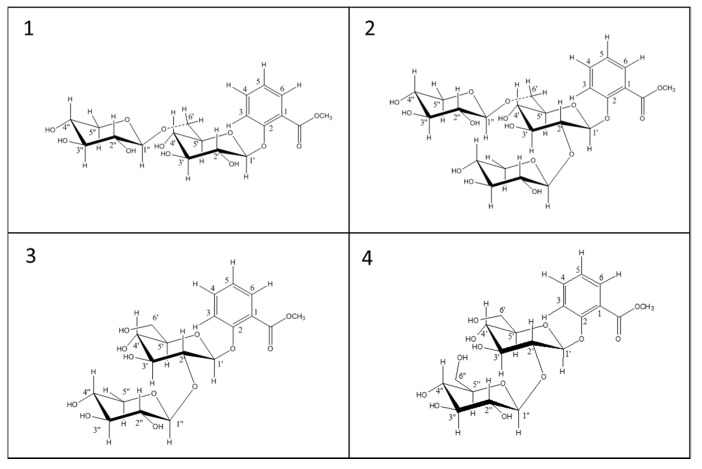
Structural formulas of the compounds isolated from the dried leaves of *Gaultheria procumbens l*. MeSA β-d-xylosyl-(1→6)-β-d-glucose (gaultherin) (**1**), MeSA xyloyl-[1→6]-xylosyl-[1→2]-glucose (MeSA-MSTG-A) (**2**), MeSA β-d-xylosyl-(1→2)-β-d-glucose (sambubiose) (**3**), MeSA β-d-glucosyl-(1→2)-β-d-glucose (sophorose) (**4**).

The wine extract also presented 1 diglycoside with mass ([M + Na]^+^ peak at *m*/*z* 483.1479 Da). The only diglycoside with this mass, reported in literature, is MeSA-rutinoside (glucose + rhamnose); for confirmation we found this compound in the methanolic extract of *Passiflora edulis* [24], which matched the peak at *m*/*z* 483.1479 Da in the wine chromatogram (Figure 2). We also found a diglycoside *m*/*z* 499.1428 (M + Na)^+^ in the wine extract. From the literature, this diglycoside could be one of the two reported diglucoside isomers *m*/*z* 499.1428 (M + Na)^+^, i.e., MeSA gentiobioside (glucose + glucose) and/or MeSA lactoside (glucose + galactose) (Figure 3). Several studies have shown that MeSA-lactoside was isolated from *Gaultheria yunnanensis (Franch.) Rehder* [25,26]. We therefore tried to inject an extract of this plant in order to identify which isomer corresponds to MeSA-lactoside. The injection excluded the presence of this compound in the wine and, therefore, the MeSA-gentiobioside isomer could be the other compound present in the wine. A gentiobioside diglycoside (Syringyl-6-*O*-β-d-glucosyl-β-d-glucopyranoside) was also reported in wine [12]. As no commercial standards are available for most MeSA glycosides, we tried to isolate some of them from *Gaultheria procumbens* L. leaves. The extraction and isolation from this plant made it possible to obtain four metabolites (Figure 4). The first compound (**1**) was confirmed by NMR analysis as gaultherin (methyl salicylate 2-*O*-β-d-xylopyranosyl (1→6) β-d-glucopyranoside). This compound is the major MeSA glycoside in *Gaultheria procumbens* plants. The isolation yielded 280 mg of (**1**) with a purity >99%.

^1^H-NMR (**1**, 400 MHz, Methanol-d_4_): 7.76 (ddd, *J* = 7.8, 1.8, 0.3 Hz, H-6, 1H), 7.57 (ddd, *J* = 8.4, 7.3, 1.8 Hz, H-4, 1H), 7.45 (ddd, *J* = 8.3, 1.1, 0.3 Hz, H-3, 1H), 7.13 (ddd, 7.8, 7.3, 1.1 Hz, H-5, 1H), 4.87 (d, *J* = 7.4 Hz, H-1′, 1H), 4.33 (d, *J* = 7.4 Hz, H-1′′, 1H), 4.13 (dd, *J* = 11.7, 1.8 Hz, H-6′b, 1H) and 3.78 (dd, *J* = 11.7, 6.5 Hz, H-6’a, 1H), 3.89 (s, 3H, OMe), 3.84 (dd, *J* = 11.5, 5.3 Hz, H-5′′ eq, 1H) and 3.15 (dd, *J* = 11.5, 10.2 Hz, H-5′′ax, 1H), 3.66 (ddd, 10.2, 8.7, 5.3 Hz, H-4′′, 1H), 3.53 (dd, 7.4, 8.7 Hz, H2’,1H), 3.47 (dd, 9.7, 9.7 Hz, H-3′′, 1H), 3.45 (m, H-5′, 1H), 3.29 (dd, 8.7, 8.7 Hz, H-3′, 1H), 3.21 (dd, *J* = 9.0, 7.4 Hz, H-2′′, 1H).

^13^C-NMR (**1**, 100 MHz, Methanol-d_4_): 168.1 (–COCH3_3_), 158.2 (C-2), 135.0 (C-4), 131.7 (C-6), 123.5 (C-5), 122.0 (C-1), 118.9 (C-3), 105.2 (C-1′′), 103.6 (C-1′), 78.0 (C-3’), 77.7 (C-3′′), 77.2 (C-5′), 74.7 (C-2′), 71.2 (C-4′), 71.0 (C-4′′), 69.6 (C-6′), 66.6 (C-5′′), 52.2 (–OCH_3_).

NMR analysis confirmed that the structure of compound **2** (435 mg, purity >99%, Figure 4) was triglycoside MSTG-A (methyl salicylate 2-*O*-β-d-xylopyranosyl (1→2) [*O*-β-d-xylopyranosyl (1→6)]-*O*-β-d–glucopyranoside).

^1^H-NMR (**2**, 400 MHz, Methanol-d_4_): 7.75 (dd, *J* = 7.8, 1.7, H-6, 1H), 7.53 (ddd, *J* = 8.4, 7.4, 1.8 Hz, H-4, 1H), 7.29 (brd, *J* = 8.4, H-3, 1H), 7.06 (ddd, 8.4, 7.8,1.0 Hz, H-5, 1H), 5.21 (d, *J* = 7.4 Hz, H-1′, 1H), 4.69 (d, 7.4, H-1′′’,1H), 4.29 (d, 7.4, H-1′′, 1H), 4.09 (dd, *J* = 11.7, 1.8 Hz, H-6’b, 1H) and 3.78 (dd, *J* = 11.7, 5.2 Hz, H-6′a, 1H), 3.89 (s, 3H, OMe), 3.82 (dd, *J* = 11.5, 5.3 Hz, H-5′′’ eq, 1H) and 3.10 (dd, *J* = 11.5, 10.2 Hz, H-5′′’ax, 1H), 3.77 (dd, 7.4, 9.0, H-2’, 1H), 3.66 (ddd, 10.2, 8.7, 5.3 Hz, H-4′′, 1H), 3.58 (dd, *J* = 50, 11.3 Hz, H-5′′ eq, 1H) and 3.13 (dd, *J* = 11.5, 10.2 Hz, H-5′′ax, 1H), 3.50 (m, H2′, 1H), 3.47 (dd, 9.7, 9.7 Hz, H-3′′, 1H), 3.45 (m, H-5′, 1H), 3.34 (dd, 8.7, 8.7 Hz, H-3′, 1H), 3.26 (dd, *J* = 9.0, 7.4 Hz, H-2′′′, 1H), 3.21 (dd, *J* = 9.0, 7.4 Hz, H-2′′, 1H), 

^13^C-NMR (**2**, 100 MHz, Methanol-d_4_): 167.8 (–COCH3_3_), 157.2 (C-2), 134.7 (C-4), 132.0 (C-6), 122.3 (C-5), 121.5 (C-1), 116.3 (C-3), 105.2 (C-1′′′), 103.1 (C-1′′), 99.7 (C-1′), 83.2 (C-2′), 78.0 (C-3′), 77.7 (C-3′′ + C-3′′′), 77.2 (C-5′), 75.8 (C-2′′), 74.7 (C-2′′′), 71.2 (C-4′), 71.0 (C-4′′ + C-4′′′), 69.4 (C-6′), 66.6 (C-5′′ + C5′′′), 52.4 (–OCH_3_).

With regard to structures **3** and **4**, we soon realized by extended NMR analysis that they do not correspond to any of those reported in Mao’s review [13].

The NMR spectra compound **3** (16 mg, 60% purity, Figure 4) suggested the new structure was methyl salicylate 2-*O*-β-d-xylopyranosyl (1→2)-β-d-glucopyranoside, formally the hydrolytic product of MSGT-A after breaking the 1′′-6′ acetalic bond. The resonance of C(6′) at δ_C_ 62.1 ruled out the possibility of a 1′′-6′ connectivity of this diglycoside (δ_C_ of C(6′) expected at about 69–70 ppm for this connectivity) and instead suggested the presence of one 1′′-2′ linkage. The detected long range ^13^C-^1^H correlation (HMBC) between the acetal proton H-C(1′′) at δ_H_ 4.68 and the carbon resonance at δ_C_ 83.2 (easily attributable to the ether-carbon C(2)) firmly established this moiety. Moreover, the characteristic coupling pattern of the geminal protons at C(5′′) at δ_H_ 3.58 (eq)/3.13(ax) can only be expected by fixing an axial position for H(C4′′), thus indicating the presence of a xylopyranose and not an arabinose moiety. Extended 2D-NMR (COSY, HSQC, HMBC) measurements allowed us to assign all the ^1^H and ^13^C resonances of this compound according to structure **3**.

^1^H-NMR (**3**, 400 MHz, Methanol-d4) δ 7.74 (ddd, *J* = 7.8, 1.8, 0.3 Hz, H-6, 1H), 7.50 (ddd, *J* = 8.4, 7.3, 1.8 Hz, H-4, 1H), 7.22 (ddd, *J* = 8.3, 1.1, 0.3 Hz, H-3, 1H), 7.05 (ddd, 7.8, 7.3, 1.1 Hz, H-5, 1H), 5.22 (d, *J* = 7.4 Hz, H-1′, 1H), 4.68 (d, *J* = 7.4 Hz, H-1′′, 1H), 3.89 (s, 3H, OMe),3.87 (dd, *J* = 11.7, 1.8 Hz, H-6’b, 1H) and 3.69 (dd, *J* = 11.7, 6.5 Hz, H-6’a, 1H), 3.78 (dd, 7.4, 8.7, H-2′, 1H), 3.67 (ddd, 10.2, 8.7, 5.3 Hz, H-3’, 1H), 3.58 (dd, *J* = 11.5, 5.2 Hz, H-5′′ eq, 1H) and 3.13 (dd, *J* = 11.4, 10.1 Hz, H-5′′ax, 1H), 3.48 (m, H-4’, 1H), 3.41 (dd, 9.7, 9.7 Hz, H-4′′, 1H), 3.29 (dd, 8.7, 8.7 Hz, H-3′′, 1H), 3.21 (dd, *J* = 9.0, 7.4 Hz, H-2′′, 1H).

^13^C-NMR (3, 100 MHz, Methanol-d_4_): 167.8 (–COCH3_3_), 157.2 (C-2), 134.2 (C-4), 131.9 (C-6), 121.5 (C-5), 115.8 (C-1), 113.0 (C-3), 105.6 (C-1′′), 99.6 (C-1′), 83.2 (C-2′), 77.5 (C-3′ + C-3′′ + C-5’), 75.5 (C-2′′), 70.7 (C-4′ + C-4′′), 66.8 (C-5′′), 62.1 (C-6′), 52.3 (OMe).

The NMR spectra compound **4** (1.8 mg, 80% purity, Figure 4) indicated the methyl salicylate 2-*O*-β-D-glucopyranosyl (1→2) β-d-glucopyranoside structure. The low amount and low purity of the isolated compound **4** did not allow for extended NMR measurements, and thus our proposed structure is only based on the ^1^H-NMR analysis and comparison with NMR data of compounds **1**–**3**.

In any case, the 1′′-2′ linkage is firmly established by the close resemblance of δ_H_ and J coupling pattern of H-C(2’) of **4** (3.82 dd, 7.4, 8.7 Hz) with H-C(2′) of **2** (3.78 dd, 7.4, 8.7 Hz).

^1^H-NMR (**4**, 400 MHz, Methanol-d_4_): Partial assignment δ 7.76 (ddd, *J* = 7.8, 1.8, 0.3 Hz, H-6, 1H), 7.52 (ddd, *J* = 8.4, 7.3, 1.8 Hz, H-4, 1H), 7.25 (ddd, *J* = 8.3, 1.1, 0.3 Hz, H-3, 1H), 7.07 (ddd, 7.8, 7.3, 1.1 Hz, H-5, 1H), 5.34 (d, *J* = 7.4 Hz, H-1′, 1H), 4.82 (d, *J* = 7.4 Hz, H-1′′, 1H), 3.88 (s, 3H, OMe), 3.86 (m, H-6’b + H-6′′b, 2H) and 3.69 (m, H-6′a + H-6′′a, 2H), 3.81 (dd, 7.4, 8.7, H-2′, 1H), 3.19 (dd, *J* = 9.0, 7.4 Hz, H-2′′, 1H). Unfortunately, apart from MeSA-primeveroside, which we already had as the standard, the other compounds did not correspond with those found in the wine extract. It was also possible to exclude the presence of triglycoside MSTG-A in our wine extracts. We found two MeSA glycosides (structure **3**–**4**) which, to our knowledge, has not been reported to date.

**Figure 5:**
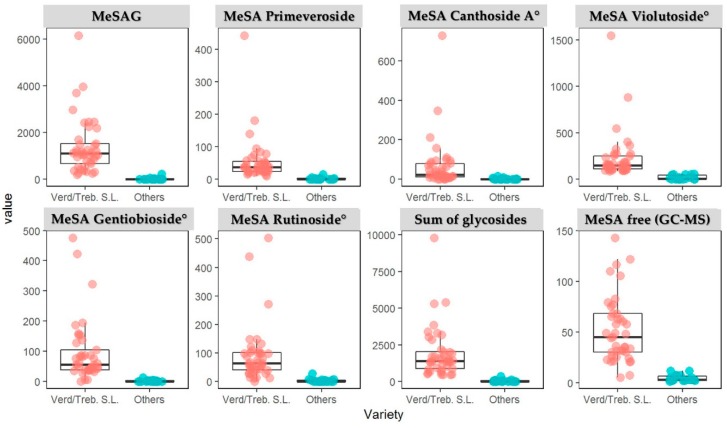
Content of MeSA-monoglycoside, MeSA-diglycosides and methyl salicylate (MeSA) in free form (value in μg/L) in Verdicchio, Trebbiano di Soave and Lugana wines compared to other Italian and international varieties. (^°^ quantified as MeSA-primeveroside).

This work was also aimed at determining whether these compounds were present in all wines or whether they were a feature of certain varieties. We therefore analyzed 64 white wines in order to understand if these glyconjugated compounds are characteristic of Verdicchio and homologous varieties (Trebbiano di Soave and Trebbiano di Lugana). We found that in the free forms the concentration of MeSA in Trebbiano di Soave/Lugana and Verdicchio varieties was in the range 5.5–143 μg/L, while the sum of the 6 glycoside precursors varied from 456–9796 μg/L (Table 2), and in “other” white wines, the free form of MeSA was less than 12 μg/L and lower than 400 μg/L as the sum of 6 glycosides. In Verdicchio/Trebbiano di Soave and Trebbiano di Lugana wines the most abundant glycoside precursor was MeSAG, followed by MeSA-vicianoside (violutoside) (Figure 5).

The sensory impact of methyl salicylate in wine is not yet particularly clear. In the literature it is described as having an odor of wintergreen, mint, and fresh green character [17], and the threshold is estimated at around 50–100 μg/L. Aroma recombination tests have to be performed by the direct omission/addition of methyl salicylate in wine in order to fully understand its importance. Some preliminary tests made by adding β-glycosidase enzymes during vinification are underway to measure how much methyl salicylate could be released by the precursors during wine aging. A balsamic note often appears when tasting old Verdicchio vintages which could stem from this compound.

## 3. Materials and Methods

### 3.1. Materials

Methyl salicylate 2-*O*-β-d-glucoside (MeSAG) and Methyl salicylate 2-*O*-β-d-xylopyranosyl (1–6) β-d-glucopyranoside (MeSA-primeveroside or Gaultherin) were from Sigma Aldrich and iChemical Technology (Shangai), respectively. Plants: *G. procumbens* (dried leaves and fruits), *G. yunnanensis (Franch.) Rehder* (dried leaves and roots), *V. cornuta V. tricolor* (flowers), *P. Ginseng* (roots), *C. Sinensis* (dried leaves) and *P. Edulis* (fruits). All solvents for GC and HPLC analysis (MS grade) were purchased from Sigma-Aldrich (Milan, Italy) Sixty-four varietal white wines were sampled from different wineries and analyzed for their content in free and bound MeSA. The samples included commercial wines produced from a pool of genetically-related Italian cultivars (Verdicchio, Trebbiano di Soave, Trebbiano di Lugana) and a group of wines from 19 diverse international and national cultivars as the comparison.

### 3.2. Solid Phase Extraction (ENV+) Procedure

10 mL of wine, added to 25 uL of *n*-heptanol (200 mg/L) as the internal standard, were extracted with solid phase extraction using ENV+ cartridges, 1 g (Biotage, Sweden). The cartridge was pre-conditioned with 15 mL of methanol followed by 20 mL of water. Then, wine was loaded onto the cartridge, which was then washed with 5 mL of water. The free methyl salicylate was eluted from the cartridge with 10 mL of dichloromethane, to which 20 mL of pentane were added. Subsequently, this fraction has been anhydrified with Na_2_SO_4_ and was carefully concentrated up to 200 μL using a Vigreux column. The bound aromatic compounds (i.e. glycosides) were eluted with 10 mL of methanol, which was eliminated under vacuum and the residue solubilized in 1 mL of methanol/water 1/9, filtered through a 0.22 µm PTFE filter into a 2 mL amber vial.

### 3.3. GC–MS Analysis of Free Methyl Salicylate

Analysis of free methyl salicylate was performed using a Trace GC Ultra gas chromatograph coupled to a Quantum XLS mass spectrometer (Thermo Scientific, Austin, TX, USA) mounted with a PAL combi-xt autosampler (CTC, Zwingen, Switzerland). A Rxi^®^-5Sil MS capillary column (30 m × 0.25 mm × 0.25 μm, Restek Corp. Bellefonte, PA) was used. One microliter of sample was injected in splitless mode with a GC inlet temperature of 250 °C. Helium was used as a carrier gas in constant flow mode at 1.2 mL/min. The oven temperature was programmed as follows: (i) initial temperature 50 °C held for 2 min, (ii) linearly raised by 8 °C/min to 150 °C, and (iii) in the final step, the temperature was ramped at 20 °C/min to 280 °C, and maintained for 1 min (total run-time was 22 min). The mass spectrometer was operated in positive electron ionization mode at 70 eV and all spectra were recorded in full scan with a mass range of 40–350 Da; transfer line and source temperatures were set at 250 °C. Electron ionization was applied at 70 eV with an emission current of 50 μA. Thermo Excalibur software (1.1.1.03, Thermo Scientific, Waltham, MA, USA) was used for all acquisition control and data processing. A calibration curve was generated, spiking the model wine solution with 5–500 μg/L of methyl salicylate.

### 3.4. UPLC-Q-TOF-HDMS for the Identification of Glycosides

An Acquity UPLC system interfaced with a Waters Synapt HDMS-QTOF mass spectrometer with electrospray ionization system (ESI) (Waters Corporation, Manchester, UK) was used to perform LC–HDMS analysis of glycosylated precursors. All samples were analyzed on a reversed phase (RP) ACQUITY UPLC 1.8 m 2.1 × 150 mm HSS T3 column (Waters) protected with an Acquity UPLC^®^ BEH HSS T3 1.8 m, 2.1 × 5 mm precolumn (Waters), at 40 °C and with a mobile phase flow rate of 0.28 mL min^−1^. Water was used as the weak eluting solvent (A) and methanol as the strong eluting solvent (B); formic acid 0.1% *v*/*v* was added in both eluents. The multistep linear gradient used was as follows: 0–1 min, 100% A isocratic; 1–3 min, 100–90% A; 3–18 min, 90–60% A; 18–21 min, 60–0% A; 21–25.5 min, 0% A isocratic; 25.5–25.6 min, 0–100% A; 25.6–28 min 100% isocratic. The injection volume was 2 µL. Mass spectrometric data were collected in positive ESI mode over a mass range of 50–2000 *m*/*z*, with a scan duration of 0.3 s in centroid mode [27].

### 3.5. UHPLC-MS/MS Ion Trap for the Quantification of Glycosides

An ExionLC system interfaced with AB6500+ QTrap mass spectrometer with electrospray ionisation system (ESI) (Applied Biosystems/MDS Sciex, Toronto, ON, Canada) was used to perform an LC–MS/MS analysis of glycosylated precursors. All samples were analyzed on a reversed phase (RP) ACQUITY UPLC 1.8 m 2.1 × 150 mm HSS T3 column (Waters) protected with an Acquity UPLC^®^ BEH HSS T3 1.8 m, 2.1 × 5 mm precolumn (Waters), at 40 °C and with a mobile phase flow rate of 0.28 mLmin^-1^. Water was used as the weak eluting solvent (A) and methanol as the strong eluting solvent (B); formic acid 0.1% *v*/*v* was added in both eluents. The multistep linear gradient used was as follows: 0–1 min, 100% A isocratic; 1–3 min, 100–90% A; 3–18 min, 90–60% A; 18–21 min, 60–0% A; 21–25.5 min, 0% A isocratic; 25.5–25.6 min, 0–100% A; 25.6–28 min 100% isocratic. Injection volume was 2 µL [24]. The transitions and spectrometric parameters were optimized individually for each standard by the direct infusion of their solutions (10 µg mL^−1^). The two most abundant fragments, to be used as the quantifier and qualifier, were identified for each compound. Declustering potential (DP) and entrance potential (EP) were optimized for each precursor ion and collision energy (CE) and Collision CellExitPotential (CXP) for each production. Table 3 shows the compound-specific instrumental parameters used in the analytical method. The presence of our metabolite of interest was confirmed using the q/Q ratio. The spray voltage was set at 5500 V for positive mode. The source temperature was set at 500 °C, the nebulizer gas (Gas1) and heater gas (Gas2) at 55 and 65 psi, respectively. The spectra derived from the precursor ion’s fragmentation were acquired in trap (enhanced product ion) to confirm the compound with a collision energy of 35 V. The two commercial standards methyl salicylate 2-*O*-β-d-glucoside and gaultherin (MeSA-primeveroside) and the isolated standard (MSTG-A) were used for the quantification. The isomers (violutoside and canthoside-A) and rutinoside and gentobioside were quantificated as equivalents of gaultherin. Samples were analyzed after filtration on 0.2 μm PTFE, injected directly and again after dilution one to hundred. Each sample was injected in triplicate. The following sodiate masses were used for quantification: 337.09 for methyl salicylate 2-*O*-β-d-glucoside, *m*/*z* 469.13 for the 3 isomers gaultherin, Canthoside A and MeSA-vicianoside (violutoside), *m*/*z* 499.14 for MeSA-gentiobioside and *m*/*z* 483.15 for MeSA-rutinoside. Calibration curves were prepared in solvent using 15 levels of MeSA 2-*O*-β-d-glucoside and gaultherin with concentrations between 0.05 µg L^−1^–and 200 µg L^−1^ (Table 3 and Table 4).

### 3.6. Isolation of Glycosides from Gaultheria Procumbens L. Dry Leaves

500 g of *Gaultheria procumbens* L. dry leaves (A. Minardi & Sons, Ravenna, Italy) were placed twice in 2 L of hot milliQ water (90 °C) and infused for 4 hours (room temperature), then centrifuged and filtered through a 0.44 µm filter.

### 3.7. Flash Chromatography with Isolute ENV+ and Preparative HPLC for the Isolation of the Single Glycosides

A Shimadzu SCL-10 AVP preparative HPLC system with a Shimadzu SPD-10 AVP UV-VIS detector, 8A pumps, and Software Class VP (Shimadzu Corp., Kyoto, Japan) were used for chromatographic separation. For flash chromatography, 30 g of ENV+ resin (Biotage, Uppsala, Sweden) were conditioned with 300 mL of methanol and 500 mL of milliQ water. 500 mL of water extract were loaded onto each batch and elution was carried out using 15 mL min^−1^ of water as solvent A and methanol as solvent B. The linear gradient went from 0 to 100% of solvent B in 120 min. The partially purified fraction with peaks of interest was collected and concentrated to 100 mL. A second separation of the partially purified fraction was done by HPLC, using a semi-preparative column Develosil^®^ 100DIOL-5 300 × 20.0 mm (CPS ANALITICA, Milan, Italy). The mobile phase was acetonitrile as solvent A and methanol 3% milliQ water (Solvent B). Run temperature was set to 65 °C, 0–15 min 0% B, 15–30 min linear gradient, 0 to 15% of B; 30–40 min 15–100% B; 40–50 min 100% B. The injection volume was 2 mL. The partially purified fraction with the peaks of interest was collected and concentrated to 20 mL. Each peak of interest derived from the Diol column was finally injected into a HPLC-DAD Alliance 2695 (Waters, Milford, MA, USA) with a Discovery HS-C18 25 cm × 10 mm, 10 µm (SUPELCO, USA) column and a UV-VIS 2996 (Waters, Manchester, UK) detector operating at 280 nm. The injection volume was 100 µL. Mobile phase milliQ water (Solvent A) and methanol (Solvent B). Run temperature was set to 40 °C, 0–2 min 5–20% B; 2–20 min 32.5%; B wash with 100% B for 2 min. Peaks of interest were fractionated with a Waters Fraction Collector 3 (Waters, Milford, MA, USA).

### 3.8. NMR Analysis

^1^H (400 MHz) and ^13^C (100 MHz) NMR spectra of the purified metabolites isolated in this study were recorded in d_4_-methanol at 300 K on a Bruker Avance 400 MHz NMR spectrometer, using a 5 mm BBI probe with 90° proton pulse length of 10.1 μs at a transmission power of 0 db and equipped with pulsed gradient field utility. The chemical shift scale (δ) was calibrated on the residual signal of deuterated methanol at δ_H_ 3.31 and δ_C_ 49.00. The following NMR experiments were carried out: ^1^H-NMR; ^1^H-^1^H COSY; ^1^H-^13^C HSQC; ^1^H-^13^C HMBC.

## 4. Conclusions

Methyl salicylate is a very intriguing molecule, both in sensorial terms and as the role it plays in plant defense and human health. This study proved the presence of MeSA in bound form in superior concentration as a distinctive characteristic of Verdicchio, Trebbiano di Soave and Trebbiano di Lugana wines. Six different MeSA precursors were found in this variety of wine, some of them reported here for the first time. These MeSA glycosides were also quantified for the first time, highlighting that MeSAG and MeSA-violutoside are the two most abundant MeSA glycosides in wine. The superior concentration of MeSA glycosides in this group of genetically-related Italian cultivars, in respect to a group of other white wines, suggests that the concentration of these compounds could be exploited in the future to trace the cultivar in commercial wines. The free MeSA released by precursors during aging could contribute toward explaining the balsamic aroma often perceived in old Verdicchio vintages, which is much sought after by winemakers and wine lovers. The analysis of the MeSA glycoside concentration in young Verdicchio wines, or possibly in the grapes, could be exploited in the future to select the grapes/wines having the potential to generate free MeSA during aging. The preparative isolation and NMR analysis of *Gaultheria procumbens* L. dry leaf extracts also allowed us to propose two novel MeSA glycoside structures.

## Figures and Tables

**Table 1:** Extract of different plants rich in certain glycosides were used for the comparison of glycosides present in Verdicchio wines.

		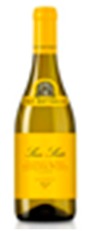	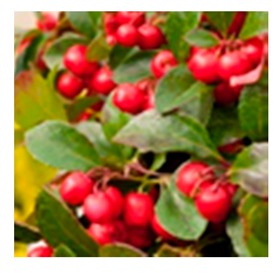	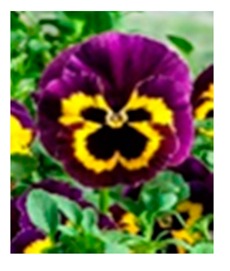	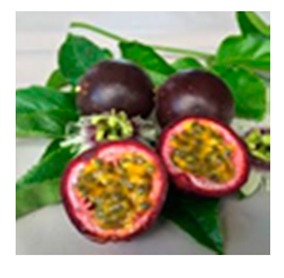	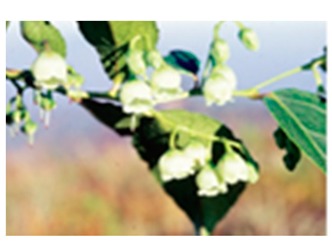
Chemical name	Common Name	Wine	*Gaultheria procumbens*	*Viola* sp.	*Passiflora edulis*	*Gaultheria yunnanensis*
methyl salicylate 2-*O*-β-d-glucoside	MeSAG	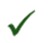	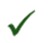	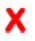	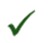	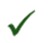
methyl salicylate 2-*O*-β-d-xylopyranosyl (1-6) β-d-glucopyranoside	MeSA-primeveroside or gaultherin	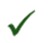	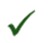	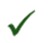	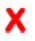	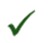
methyl salicylate 2-*O*-α-l-arabinopyranosyl (1-6)-β-d-glucopyranoside	MeSA-vicianoside or violutoside	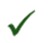	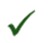	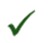	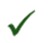	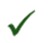
methyl salicylate 2-*O*-β-d-apiofuranosyl (1-6)-β-d-glucopyranoside	MeSA-canthoside A	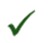	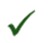	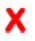	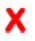	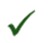
methyl salicylate 2-*O*-β-d-galactopyranosyl (1-4)-β-d-glucopyranoside	MeSA-lactoside	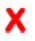	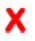	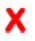	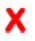	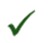
methyl salicylate 2-*O*-β-d-glucopyranosyl (1-6)-*O*-β-d-glucopyranoside	MeSA-gentiobioside	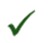	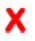	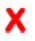	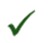	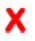
methyl salicylate 2-*O*-α-l-rhamnopyranosyl (1-6)-β-d-glucopyranoside	MeSA-rutinoside	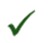	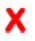	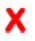	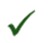	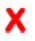
methyl salicylate 2-*O*-β-d-xylopyranosyl (1→2)-β-d-glucopyranoside	MeSA-sambubioside	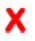	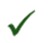	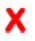	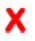	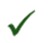
methyl salicylate 2-*O*-β-d-glucopyranosyl (1→2)β-d-glucopyranoside	MeSA-sophoroside	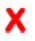	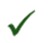	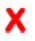	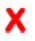	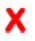
methyl salicylate 2-*O*-β-d-xylopyranosyl (1-2)[*O*-β-d-xylopyranosyl(1-6)]-*O*-β-d-glucopyranoside	MSTG-A	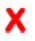	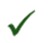	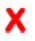	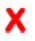	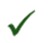
methyl salicylate 2-*O*-β-d-glucopyranosyl (1-2)[O-β-d-xylopyranosyl(1-6)]-*O*-β-d-glucopyranoside	MSTG-B	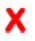	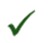	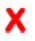	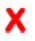	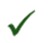

**Table 2:** Concentration (means ± standard deviation) of different MeSA glycosides and free MeSA in different wine varieties.

Variety	Vintage	Code	Glycosides							Free
			Monoglycosides	Diglycosides					Sum of All	
			MeSAG	MeSA-Primeveroside	MeSA-Canthoside A ^a^	MeSA-Violutoside ^a^	MeSA-Gentiobioside ^a^	MeSA-Rutinoside ^a^		MeSA
Verdicchio	2008	Verd/Treb. S.L.	2455 ± 369	57.1 ± 2.1	<0.1	275 ± 6.3	476 ± 36.0	39.4 ± 0.9	3303 ± 389	83.1 ± 5.1
Verdicchio	2010	Verd/Treb. S.L.	2427 ± 467	78.1 ± 3.8	38.2 ± 3.3	253 ± 15.9	323 ± 36.1	272 ± 10.4	3391 ± 487	63.5 ± 2.9
Verdicchio	2013	Verd/Treb. S.L.	187 ± 29.7	22.8 ± 2.5	5.5 ± 0.3	113 ± 2.8	187 ± 8.2	<0.1	515 ± 33.3	21.3 ± 1.9
Verdicchio	2014	Verd/Treb. S.L.	244 ± 28.1	21.8 ± 1.1	18.4 ± 0.7	98.6 ± 2.3	82.6 ± 4.9	14.1 ± 0.3	479 ± 20.3	5.5 ± 0.3
Verdicchio	2015	Verd/Treb. S.L.	520 ± 57.7	23.1 ± 1.2	8.8 ± 0.2	92.9 ± 1.2	48.3 ± 1.8	75.4 ± 2.6	769 ± 56.9	24 ± 0.2
Verdicchio	2016	Verd/Treb. S.L.	1141 ± 145	38.7 ± 2.1	21.8 ± 2.2	148 ± 7	87 ± 1.8	98.3 ± 4.1	1535 ± 157	49.2 ± 0.7
Verdicchio	2016	Verd/Treb. S.L.	785 ± 125	26.7 ± 2.1	10.6 ± 0.2	137 ± 4.4	42.9 ± 1.9	44.1 ± 2.0	1046 ± 135	48.5 ± 0.9
Verdicchio	2016	Verd/Treb. S.L.	531 ± 74.8	32.9 ± 1.3	38.3 ± 0.3	172 ± 9.3	36.8 ± 1.5	11.6 ± 0.6	823 ± 83.5	25.4 ± 2.1
Verdicchio	2016	Verd/Treb. S.L.	2243 ± 213	75.7 ± 2.5	112 ± 1.3	403 ± 54.8	155 ± 5.6	41.4 ± 1.6	3030 ± 267	44.3 ± 1.8
Verdicchio	2016	Verd/Treb. S.L.	749 ± 103	18.9 ± 0.8	16.7 ± 1.3	99.7 ± 3.7	36.3 ± 1.9	27.9 ± 6.3	949 ± 109	30.3 ± 0.9
Verdicchio	2016	Verd/Treb. S.L.	872 ± 67	23.9 ± 1.0	24.4 ± 0.8	110 ± 3.9	37.3 ± 1.2	57.5 ± 2.0	1125 ± 67.4	26.2 ± 0.2
Verdicchio	2016	Verd/Treb. S.L.	955 ± 124	37.9 ± 0.8	14.3 ± 1.4	137.3 ± 5.4	128 ± 5.5	62.1 ± 0.6	1335 ± 111	21.6 ± 2.4
Verdicchio	2017	Verd/Treb. S.L.	1120 ± 99	9.9 ± 0.6	<0.1	157 ± 5.6	0.8 ± 0.1	67.8 ± 1.8	1356 ± 94	122 ± 9.1
Verdicchio	2017	Verd/Treb. S.L.	990 ± 123	40.2 ± 1.6	22.9 ± 0.5	147 ± 5.4	86.8 ± 2.6	98.7 ± 2.7	1386 ± 128	57.5 ± 0.8
Verdicchio	2017	Verd/Treb. S.L.	1033 ± 108	43.3 ± 0.1	23.6 ± 0.4	152 ± 8.3	83.4 ± 8.0	101 ± 2.8	1436 ± 95	57.1 ± 1.7
Verdicchio	2018	Verd/Treb. S.L.	1178 ± 217	33.8 ± 1.6	63.2 ± 3.7	128 ± 4.8	36.5 ± 1.2	82.6 ± 3.0	1522 ± 229	65.3 ± 2.1
Verdicchio	2018	Verd/Treb. S.L.	1151.5 ± 97	42.7 ± 2.9	78.4 ± 9.5	161 ± 11.1	40.7 ± 1.9	106 ± 9.5	1580 ± 102	35.2 ± 3.2
Verdicchio	2018	Verd/Treb. S.L.	3700 ± 309	181 ± 15.3	348 ± 5.6	545 ± 11.4	87.1 ± 6.3	438 ± 34.0	5299 ± 333	117 ± 9.6
Verdicchio	2018	Verd/Treb. S.L.	2183 ± 228	83.3 ± 25.4	95.3 ± 36.5	273 ± 13.0	60.1 ± 16.1	149 ± 16.5	2844 ± 312	77.5 ± 0.5
Verdicchio	2018	Verd/Treb. S.L.	2456 ± 224	66.9 ± 16.6	102 ± 42.1	363 ± 39.6	57.1 ± 9.4	133 ± 26.8	3178 ± 349	75.5 ± 2.6
Verdicchio	2018	Verd/Treb. S.L.	1267 ± 95.8	43.6 ± 9.3	94.4 ± 18.2	152 ± 15.9	54.7 ± 10.9	101 ± 23.2	1713 ± 170	36.5 ± 0.4
Verdicchio	2018	Verd/Treb. S.L.	1485 ± 44.8	55.2 ± 4.8	89.3 ± 11.5	166 ± 7.4	104 ± 11.2	122 ± 12.1	2022 ± 83.2	60.5 ± 3.2
Verdicchio	2018	Verd/Treb. S.L.	1058 ± 84.8	26.1 ± 13.6	48.4 ± 17.4	107 ± 31.1	32.8 ± 9.8	65.2 ± 19.9	1338 ± 170	62.8 ± 6.2
Verdicchio	2018	Verd/Treb. S.L.	1151 ± 97	42.7 ± 2.9	78.4 ± 9.5	161 ± 11.1	40.7 ± 1.9	106 ± 9.5	1580 ± 102	35.1 ± 1.2
Verdicchio	2018	Verd/Treb. S.L.	1518 ± 120	27.4 ± 5.7	48.7 ± 7.8	276 ± 24.3	60.2 ± 3.1	89.7 ± 2.7	2020 ± 130	31.5 ± 2.1
Verdicchio	2018	Verd/Treb. S.L.	1451 ± 84.8	46.8 ± 1.3	82.5 ± 5.8	187 ± 12.9	47.3 ± 4.1	98.6 ± 3.9	1913 ± 86.5	106 ± 12.1
Verdicchio	2018	Verd/Treb. S.L.	1694 ± 110	49.8 ± 1.9	71.1 ± 5.8	185 ± 16.2	47.7 ± 3.4	148.7 ± 8.1	2196 ± 106	79.7 ± 5.8
Verdicchio	2018	Verd/Treb. S.L.	6150 ± 330	443 ± 46.0	729 ± 32.6	1548 ± 16.6	423 ± 9.6	503 ± 27.5	9796 ± 398	143 ± 11.2
Verdicchio	2018	Verd/Treb. S.L.	1021 ± 89.2	37.1 ± 1.5	61.8 ± 9.5	142 ± 11.8	44.2 ± 7.2	98.2 ± 2.7	1404 ± 89.7	49.2 ± 6.3
Verdicchio	2018	Verd/Treb. S.L.	1058 ± 120	26.1 ± 13.6	48.4 ± 7.8	107 ± 31.1	32.8 ± 9.8	65.2 ± 19.9	1338 ± 86.8	62.8 ± 6.9
Verdicchio	2018	Verd/Treb. S.L.	375 ± 48.7	23.3 ± 0.9	10.3 ± 0.9	109 ± 6.5	38.3 ± 1.5	40.8 ± 0.7	597 ± 55.0	20.6 ± 0.3
Verdicchio	2018	Verd/Treb. S.L.	667 ± 58.6	21.3 ± 1.1	18.3 ± 0.9	98.6 ± 1.3	28.9 ± 1.1	53.6 ± 3.6	888 ± 64.2	30.3 ± 1.2
Verdicchio	2018	Verd/Treb. S.L.	423 ± 50.3	25.6 ± 0.7	12 ± 0.5	110 ± 3.5	39.5 ± 1.8	55.2 ± 2.3	665 ± 56.4	23 ± 0.5
Trebbiano di Soave	2016	Verd/Treb. S.L.	3966 ± 742	140 ± 32.2	212 ± 48.3	882 ± 90.7	153 ± 4.1	41.9 ± 1.8	5395 ± 870	45.4 ± 2.5
Trebbiano di Soave	2016	Verd/Treb. S.L.	369 ± 70.4	25.3 ± 0.6	17.4 ± 0.8	163 ± 3.6	39.1 ± 1.4	14.7 ± 0.6	629 ± 70.6	21 ± 1.2
Trebbiano di Soave	2017	Verd/Treb. S.L.	262 ± 41.1	23.6 ± 1.1	16.3 ± 0.2	120 ± 2.8	46.5 ± 0.5	13.5 ± 0.1	482 ± 45.3	7.5 ± 0.5
Trebbiano di Soave	2017	Verd/Treb. S.L.	319 ± 41.1	15.6 ± 0.5	<0.1	87.5 ± 2.5	5.8 ± 0.3	28.2 ± 1.4	456 ± 42.5	68.6 ± 3.2
Trebbiano di Soave	2017	Verd/Treb. S.L.	317 ± 25.4	14.6 ± 0.7	<0.1	89.9 ± 4.1	5.7 ± 0.4	28.9 ± 1.4	456 ± 20.3	68.6 ± 3.1
Trebbiano di Lugana	2016	Verd/Treb. S.L.	2960 ± 366	95.3 ± 2.5	158 ± 3.4	329 ± 28.7	194 ± 1.2	109 ± 3.4	3845 ± 361	110 ± 2.3
Trebbiano di Lugana	2016	Verd/Treb. S.L.	1227 ± 126	50.5 ± 2.1	23.2 ± 0.9	234 ± 7.9	137 ± 4.3	28.3 ± 1.7	1700 ± 109	31.7 ± 1.8
Trebbiano di Lugana	2016	Verd/Treb. S.L.	1244 ± 108	58.4 ± 1.4	23.8 ± 1.2	250 ± 10.8	159 ± 5.6	64.1 ± 1.8	1799 ± 93	35.6 ± 1.8
Trebbiano di Lugana	2017	Verd/Treb. S.L.	1097 ± 105	37.5 ± 1.8	6.8 ± 0.9	97.9 ± 0.9	76.6 ± 5.0	54.1 ± 0.5	1370 ± 106	32 ± 0.9
Trebbiano di Lugana	2017	Verd/Treb. S.L.	1028 ± 81.7	36.7 ± 1.3	6.3 ± 1.1	95.9 ± 5.4	77 ± 1.8	54.6 ± 0.4	1299 ± 72.5	34.0 ± 1.6
Bianca	2016	Others	24.5 ± 9.9	<0.1	<0.1	20.3 ± 0.4	3.3 ± 0.2	5.8 ± 0.6	53.9 ± 9.7	12 ± 1.1
Trebbiano Abruzzo	2014	Others	37.5 ± 0.9	4.5 ± 0.4	<0.1	59.4 ± 1.1	5.3 ± 0.1	3.6 ± 0.9	110 ± 1.6	2.1 ± 0.01
Peverella	2013	Others	8.8 ± 0.4	5.5 ± 0.2	4.7 ± 0.6	44.3 ± 2.1	<0.1	<0.1	63.3 ± 2.9	5.6 ± 0.8
Riesling Renano	2016	Others	6.8 ± 0.2	<0.1	<0.1	12.5 ± 0.6	1.8 ± 0.3	5.8 ± 1.1	26.9 ± 1.0	3.1 ± 0.01
Helios	2016	Others	10.7 ± 0.8	<0.1	6.6 ± 1.0	53.8 ± 0.5	2.6 ± 0.4	5.6 ± 0.8	79.3 ± 2.4	2.1 ± 0.02
Moscato d’Asti	2010	Others	7.3 ± 0.3	<0.1	<0.1	21.2 ± 2.2	3.0 ± 0.2	4.9 ± 0.5	36.4 ± 2.0	3.2 ± 0.01
Trebbiano Abruzzo	2012	Others	29 ± 9.6	2.6 ± 0.3	<0.1	46.7 ± 1.4	2.6 ± 0.6	8.3 ± 0.1	89.2 ± 8.1	1.3 ± 0.05
Müller Thurgau	2016	Others	7.5 ± 0.4	<0.1	<0.1	6.1 ± 0.8	<0.1	<0.1	13.6 ± 0.9	5.2 ± 0.3
Grüner Veltliner	2009	Others	4.2 ± 0.2	<0.1	<0.1	8.1 ± 0.5	<0.1	<0.1	12.3 ± 0.7	2.1 ± 0.1
Sauvignon Blanc	2011	Others	62.4 ± 11.8	<0.1	<0.1	52.6 ± 2.1	5.2 ± 0.6	2.3 ± 1.1	123 ± 14.7	3.2 ± 0.04
Pinot grigio	2015	Others	8.2 ± 0.2	<0.1	<0.1	32.1 ± 1.7	1.2 ± 0.3	7.7 ± 0.3	49.2 ± 2.0	3.1 ± 0.05
Chardonnay	2016	Others	4.9 ± 0.3	<0.1	2.8 ± 0.02	<0.1	<0.1	<0.1	7.7 ± 0.9	7.4 ± 1.0
Cataratto	2016	Others	236 ± 11.7	15.5 ± 0.4	17.2 ± 0.9	52.5 ± 1.4	14 ± 0.3	28.2 ± 2.2	363 ± 10.3	7.7 ± 0.6
Friulano	2012	Others	9.0 ± 0.6	<0.1	<0.1	5.4 ± 0.4	<0.1	6.3 ± 0.4	20.7 ± 0.9	3.0 ± 0.05
Ribolla gialla	2016	Others	1.5 ± 0.01	<0.1	0.5 ± 0.02	<0.1	<0.1	<0.1	2.0 ± 0.06	2.1 ± 0.06
Riesling renano	2014	Others	2.6 ± 0.04	2.5 ± 0.04	1.4 ± 0.01	<0.1	<0.1	<0.1	6.5 ± 1.6	12.0 ± 0.9
Pinot grigio	2015	Others	<0.05	<0.1	0.2 ± 0.02	<0.1	<0.1	<0.1	0.2 ± 0.02	2.9 ± 0.01
Psarades	2016	Others	5.1 ± 0.06	<0.1	2.5 ± 0.01	<0.1	<0.1	<0.1	7.6 ± 0.5	7.7 ± 0.6
Baiano	2017	Others	0.7 ± 0.01	<0.1	8.9 ± 0.5	<0.1	<0.1	<0.1	9.6 ± 0.4	2.1 ± 0.01
Muscaris	2017	Others	<0.05	2.5 ± 0.01	0.1	<0.1	<0.1	<0.1	2.5 ± 0.3	5.2 ± 0.4
Gewürztraminer	2009	Others	<0.05	<0.1	<0.1	<0.1	<0.1	<0.1	<0.1	4.0 ± 0.3

Concentration expressed in μg/L (^a^ quantified as MeSA-primeveroside).

**Table 3:** Chemical name, common name, molecular formula and instrument parameters for the LC-MS/MS quantification of different MeSA glycosides.

Name	Common Name	Molecular Formula	Ionization Mode	Precursor Ion	Q1 Product Ion	DP	EP	CE	CXP	Q2 Product Ion	DP	EP	CE	CXP	t_R_	Q2/Q1
methyl salicylate 2-*O*-β-d-glucoside	MeSAG	C_14_H_18_O_8_	[M + Na]^+^	337.0	337.0	80	10	10	15	185.2	80	10	24	15	15.6	17
methyl salicylate 2-*O*-α-l-arabinopyranosyl(1→6)-β-d-glucopyranoside	MeSA-Violutoside/Vicianoside	C_19_H_26_O_12_	[M + Na]^+^	469.1	469.1	80	10	10	15	337.2	80	10	38	15	15.2	108
methyl salicylate 2-*O*-β-d-xylopyranosyl (1→6)-β-d-glucopyranoside	MeSA-Primeveroside/Gaultherin	C_19_H_26_O_12_	[M + Na]^+^	469.1	469.1	80	10	10	15	337.2	80	10	38	15	15.7	108
methyl salicylate 2-*O*-β-d-apiofuranosyl(1→6)-β-d-glucopyranoside	MeSA-Canthoside A	C_19_H_26_O_12_	[M + Na]^+^	469.1	469.1	80	10	10	15	337.2	80	10	38	15	14.9	108
methyl salicylate 2-*O*-α-l-rhamnopyranosyl(1→6)-β-d-glucopyranoside	MeSA-rutinoside	C_20_H_28_O_12_	[M + Na]^+^	483.1	483.1	80	10	10	15	337.1	80	10	35	15	16.8	60
methyl salicylate 2-*O*-β-d-glucopyranosyl(1→6)-*O*-β-d-glucopyranoside	MeSA-gentiobioside	C_20_H_28_O_13_	[M + Na]^+^	499.1	499.1	80	10	10	15	347.2	80	10	31	15	13.6	20
methyl salicylate 2-*O*-β-d-xylopyranosyl (1→2)[*O*-β-d-xylopyranosyl(1→6)]-*O*-β-d-glucopyranoside	MSTG-A	C_24_H_34_O_16_	[M + Na]^+^	601.1	601.1	80	10	10	15	449.2	80	10	38	15	14.5	14

**Table 4:** Quantification parameters.

Name	Common Name	Linearity Range (µg/L)	Solvent Calibration Curves Equation	R^2^	LOQ (µg/L)
methyl salicylate 2-*O*-β-d-glucoside	MeSAG	0.05–200	Y = 1377830x + 268124	0.99	0.05
methyl salicylate 2-*O*-β-d-xylopyranosyl (1→6)-β-d-glucopyranoside	MeSA-primeveroside/gaultherin	0.1–200	Y = 1671150x + 2311990	0.98	0.1
methyl salicylate 2-*O*-β-d-xylopyranosyl (1→2)[*O*-β-d-xylopyranosyl(1→6)]-*O*-β-d-glucopyranoside	MSTG-A	0.1–200	Y = 603915x + 269546	0.99	0.1
